# Supplementary material for: Fresh keeping decision and coordination of fresh agricultural products supply chain under carbon cap-and-trade
Source: PLoS One. 2023 Apr 3;18(4):e0283872. doi: 10.1371/journal.pone.0283872 (PMC10069793; doi:10.1371/journal.pone.0283872)
Supplement: S1 Dataset — (DOCX) [file pone.0283872.s001.docx]

**Data Set**

In this paper, we use game theory to study the fresh-keeping decision and coordination contract design of the supplier-led two-echelon fresh agricultural product supply chain under the carbon cap-and-trade. Readers can replicate the results of our study through the proofs in the article.

We also use numerical example in this paper. The original data of parameters are: $d=50$, $b=1$, $c=10$, $k=2$, $\mu=4$, $n=2$, $\gamma=2$, $e=0.5$, $\beta=1$, $E=40$. Substituting these data to equations we obtained, the readers can redraw the figures in this paper.
